# Distribution, Habitat Use and Conservation of the Bornean Ferret Badger

**DOI:** 10.1002/ece3.73756

**Published:** 2026-06-03

**Authors:** Andrew J. Hearn, Mohammad Aliyuddin bin Jaini, Caroline Charão Sartor, Pg Mohd Sahlan bin Salam, Andy Martin, Chrishen Gomez, David W. Macdonald

**Affiliations:** ^1^ Wildlife Conservation Research Unit, Department of Biology University of Oxford, Life and Mind Building Oxford UK; ^2^ Sabah Forestry Department Forest Research Centre Sandakan Sabah Malaysia; ^3^ Research & Education Division Kota Kinabalu Sabah Malaysia

**Keywords:** habitat suitability modelling, landscape connectivity, *Melogale everetti*, montane forest conservation

## Abstract

The Bornean ferret badger ( *Melogale everetti* ) is one of Southeast Asia's most geographically restricted carnivores and is listed as Endangered, yet its ecology, distribution and conservation needs remain poorly understood. We conducted multi‐site camera‐trap surveys across Sabah's western mountain massif—the species' only confirmed area of occurrence—and integrated these data with multi‐scale habitat suitability modelling and landscape connectivity analyses to refine its probable range, habitat associations and movement potential. Surveys yielded 407 independent detections from 60 camera stations, including the first confirmed records from Nuluhon–Trusmadi Forest Reserve, extending the verified distribution eastward beyond the Kinabalu–Crocker axis. Estimated detection probability per sampling occasion was generally low, but occupancy modelling indicated widespread site use, with highest model‐based occupancy in Tenompok Forest Reserve and a mixed farmland site, and similarly high values in adjacent Kinabalu Ecolinc and southern Kinabalu Park. Habitat suitability was primarily associated with topographic position index and soil properties, suggesting edaphic and landform associations consistent with fossorial foraging ecology. The Sunda stink badger ( *Mydaus javanensis* ), an ecologically similar musteloid, was detected in only two highland areas, both of which lacked or supported very low ferret badger occurrence; this segregation is consistent with (but not definitive evidence for) interspecific competition. Connectivity analyses revealed contiguous Bornean ferret badger movement zones within core habitat patches but no functional corridors between them, highlighting the importance of the Kinabalu Ecolinc for demographic exchange. Model‐based estimates of Area of Occupancy (2424 km^2^) and Extent of Occurrence (4795.6 km^2^) exceed previous IUCN values but remain consistent with Endangered status. Our findings identify priority upland conservation areas, emerging barriers to connectivity, and provide a robust basis for conservation planning in Sabah's montane landscapes.

## Graphical abstract

The Bornean ferret badger ( *Melogale everetti* ) is an Endangered, range‐restricted carnivore whose ecology and distribution remain poorly known. Using multi‐site camera‐trap surveys across Sabah's western mountain massif combined with habitat suitability and connectivity modelling, we documented 407 detections from 60 stations, including the first confirmed records from Nuluhon–Trusmadi Forest Reserve, and identified strong associations with topographic position and soil properties consistent with fossorial foraging. Connectivity analyses revealed isolated core habitat patches with limited functional corridors, while updated estimates of Area of Occupancy (2424 km^2^) and Extent of Occurrence (4795.6 km^2^) exceed previous values yet support continued Endangered status, highlighting priority upland areas for conservation planning.

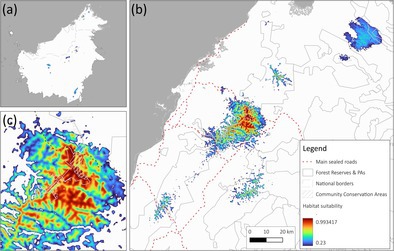

## 1 Introduction

Species within the same genus can differ markedly in ecological niche and geographic range. Some occupy diverse environments and expansive geographic areas, while others are confined to narrow habitat types or climatic conditions. This variation reflects a complex interplay of evolutionary history, biogeography, habitat heterogeneity and species interactions (Gaston 2003). Across taxa, broader environmental, habitat or dietary niches are generally associated with larger distributions (Slatyer et al. 2013). Species evolving in stable, resource‐consistent environments tend to develop narrow ecological tolerances, whereas those in variable or fragmented landscapes often display greater ecological flexibility (Brown 1984; Devictor et al. 2010). Island systems can foster both adaptability and extreme specialization, producing striking contrasts even among close relatives with similar morphology and presumed ecological roles (Whittaker et al. 2023).

The genus *Melogale*—the ferret badgers—illustrates this variation well. All species are small‐bodied (< 3 kg) mustelids with similar body plans, fossorial adaptations and invertebrate‐based diets, yet their geographic ranges differ widely. Continental species such as *M. moschata* and *M. personata* occupy diverse habitats and have extensive distributions in mainland South and Southeast Asia and southern China, respectively. Island endemics like *M. m. subaurantiaca* in Taiwan and *M. orientalis* in Java are similarly adaptable, spanning broad elevational and habitat gradients across their islands (e.g., Duckworth et al. 2016, 2024). In sharp contrast, the Bornean ferret badger ( *M. everetti* ) is confined to the western mountain massif of Sabah and remains one of Southeast Asia's least‐known Carnivora. Its absence from apparently suitable upland habitats elsewhere on Borneo suggests that, beyond environmental constraints, intraguild interactions—including exploitative or interference competition and, potentially, intraguild predation—may influence its distribution.

Despite nearly two decades of intensive camera‐trap effort across Sabah and elsewhere in Borneo, new records of the Bornean ferret badger remain scarce and confined to the western highlands of Sabah. Recent detections are from Kinabalu Park (Dinets 2003; Camacho‐Sanchez et al. 2019), Crocker Range Park (Wong et al. 2011; Ross et al. 2017), Tenompok Forest Reserve, and adjacent community‐managed lands within the Kinabalu Ecolinc corridor (Nagano et al. 2019). The species occurs from ~750 m to > 3300 m elevation and is recorded in both primary montane forest and open fields maintained under traditional slash‐and‐burn agriculture (Nagano et al. 2019). This apparent tolerance of disturbance, coupled with broad elevational occupancy, accords with the expectation that species with wider niche breadths tend to have larger distributions (Slatyer et al. 2013). Yet, despite seemingly suitable upland habitats elsewhere on Borneo, the Bornean ferret badger is unrecorded from Brunei, Sarawak or Kalimantan.

Biotic interactions within the carnivore guild may constrain the realised distribution of this carnivore. Predation is a recognised process shaping carnivore guild structure, with felids the most likely predators of the small‐bodied ferret badger. Recent camera‐trap surveys, however, show Bornean felids are positively associated with Sabah's western highlands (e.g., Hearn et al. 2025), offering little support for this as the primary limiting factor. Other carnivores may still exert predation pressure, so the hypothesis cannot be discounted entirely. Another plausible mechanism is intraguild competition with ecologically similar meso‐predators, for which the Sunda stink badger ( *Mydaus javanensis* ) is the leading candidate. This Mustelid is widespread on Borneo, similar in size and fossorial habits to the Bornean ferret badger, and shares a largely invertebrate‐based diet. It occupies a wide range of habitats, tolerates anthropogenic disturbance (Ross et al. 2017), and preliminary models suggest the western highlands may be largely unsuitable for the species (Samejima et al. 2016). These observations lead to the tentative hypothesis that Sunda stink badger's absence or low abundance in the highlands may facilitate the Bornean ferret badger's persistence there, while its widespread presence elsewhere could limit the ferret badger's range.

Range‐restricted mammals in tropical montane systems contribute disproportionately to global biodiversity yet remain among the least studied and most vulnerable taxa. Improving knowledge of their distributions, habitat requirements and landscape connectivity is essential for prioritising conservation action in regions experiencing rapid infrastructure expansion and land‐use change. The Bornean ferret badger provides a model system for examining how fine‐scale environmental filters and biotic interactions shape the persistence of endemic carnivores in human‐modified montane landscapes.

Here, we present the most comprehensive study of the Bornean ferret badger ( *Melogale everetti* ) to date, integrating new camera‐trap data from Sabah's western highlands with spatial modelling to: (1) refine its distribution and habitat associations across elevational and land‐use gradients; (2) evaluate environmental correlates of habitat suitability at multiple spatial scales, focusing on traits linked to fossorial foraging; (3) assess functional connectivity between subpopulations; (4) explore the hypothesis that its restricted range is shaped by competition with the Sunda stink badger through analysis of spatial overlap and relative abundance; and (5) identify key threats and conservation priorities.

## 2 Materials and Methods

### 2.1 Camera Trap Surveys

**FIGURE 1:**
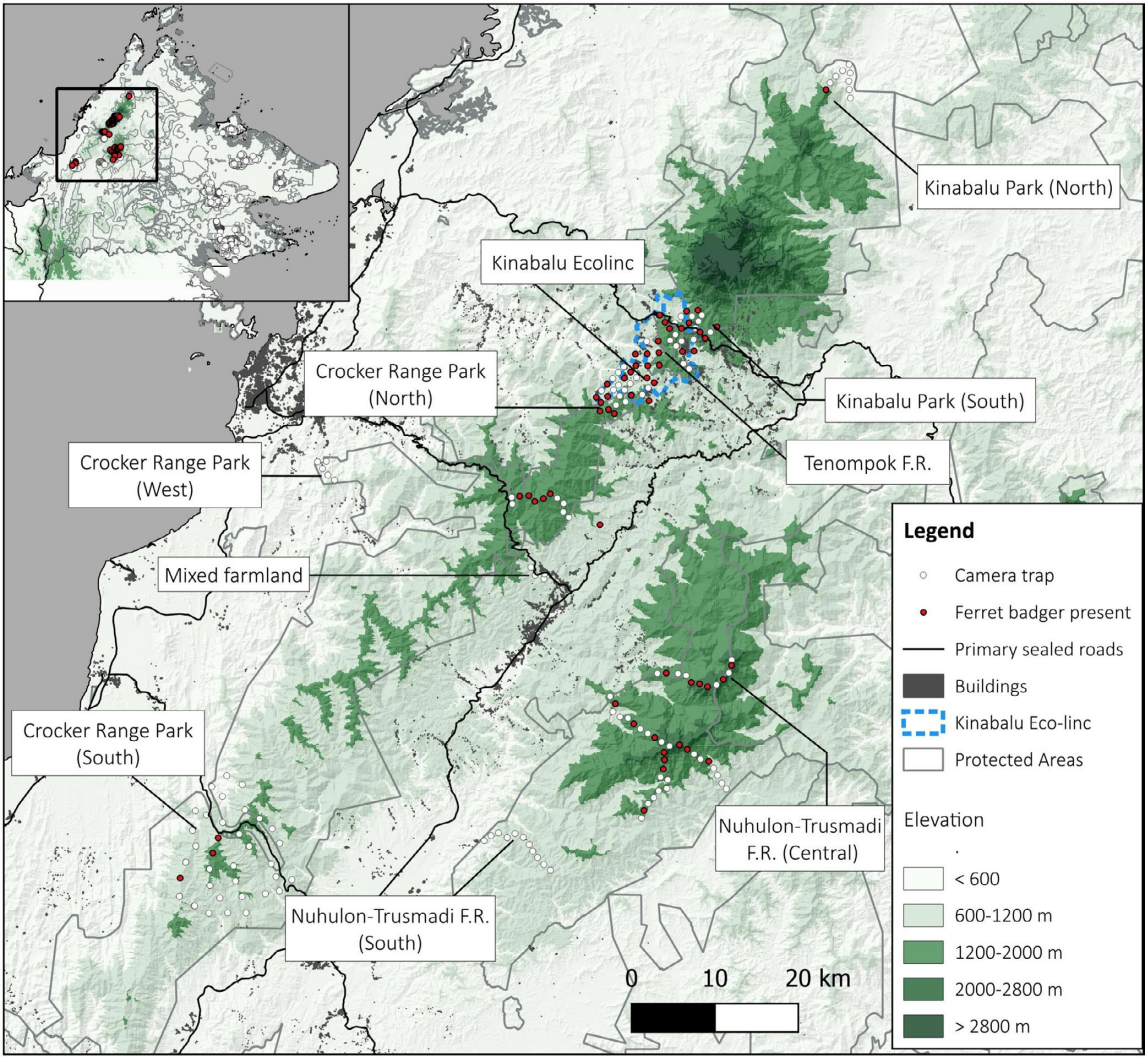
Location of camera trap surveys and Bornean ferret badger records in Sabah, Malaysian Borneo.

From December 2021 to September 2024, we conducted camera trap surveys across 10 areas in Sabah's western mountain massif (Figure 1; Table 1). This montane complex contains Borneo's three highest peaks—Gunung Kinabalu (4095 m), Gunung Trusmadi (2642 m) and Gunung Sinsing (2603 m)—and encompasses a heterogeneous mix of forest types, altitudinal gradients, and land‐use regimes. Vegetation follows a distinct elevational sequence, from lowland (< 600 m) and hill (600–1200 m) dipterocarp forests to lower (1200–2000 m) and upper (2000–2800 m) montane forests, transitioning into sub‐alpine (2800–3700 m) and alpine (> 3700 m) zones at the highest elevations (Kitayama 1992).

**TABLE 1:** Description of survey areas in Sabah's western highlands.

Region	Dominant land uses	Effort (trap nights)	Elevation (m)	
			Mean	Range
Crocker Range (North)	Primary hill dipterocarp & lower montane forest	2109	1615	1092–2017
Crocker Range (South)	Primary Lowland & Hill Dipterocarp, & Lower montane forest	4708	1068	383–1452
Crocker Range (West)	Disturbed & primary lowland & hill dipterocarp	464	534	329–751
Ecolinc (Mosaic)	Mosaic of villages, agricultural fields, burnt areas & secondary forest	3064	1057	712–1593
Kinabalu Park (North)	Primary lowland & hill dipterocarp, & lower montane forest	757	536	199–1415
Kinabalu Park (South)	Primary lower and upper montane forest	894	1605	1410–1791
Mixed farmland	Mix of perennial crops, fruit trees & oil palms	467	912	813–1042
Tenompok FR	Disturbed Lower montane forest	838	1385	1023–1650
Trusmadi FR (Central)	Primary Hill dipterocarp, lower & upper montane forest	5962	1659	690–2641
Trusmadi FR (South)	Selectively logged Hill dipterocarp forest	1107	878	567–1072

Survey locations were selected to capture variation in protection status, land‐use intensity and landscape context, allowing direct evaluation of conservation‐relevant factors influencing occurrence and connectivity. Surveys focused on Class I protected areas where the Bornean ferret badger has previously been recorded, including two regions of Kinabalu Park (754 km^2^), three in Crocker Range Park (1399 km^2^) and Tenompok Forest Reserve (20 km^2^). We also sampled within the Kinabalu Ecolinc corridor, a Sabah Parks‐led initiative establishing Community Conserved Areas to enhance connectivity between Kinabalu and Crocker Range parks. This landscape comprises a mosaic of human settlements, swidden fields and patches of recovering secondary forest. To refine distributional limits, we surveyed additional adjacent areas lacking prior *Melogale everetti* records: the Nuluhon‐Trusmadi Forest Reserve (747 km^2^; hereafter Trusmadi) and a small mixed‐use farmland patch ~7 km north of Tambunan town.

We deployed Reconyx HF2X passive infrared digital camera traps (Reconyx Inc., WI, USA) across 188 camera stations. Where logistically feasible, we aimed to cover the full elevational range of each area, with cameras spanning 199–2641 m above sea level (Figure 1; Table 1). Stations were spaced ~1.0–1.5 km apart, mounted individually at ~30 cm from the ground to optimize detection of small terrestrial fauna, and operated without bait. To reduce theft risk, cameras were set ≥ 10 m from well‐used trails and avoided active or disused logging roads, except in southern Crocker Range Park where ridgelines—also key wildlife routes—were targeted despite occasional human use (see Hearn et al. 2016). These deployment choices also aimed to minimise potential behavioural avoidance of cameras, although some influence on detection probability cannot be excluded. Consecutive photographs of the same species at a given camera station were considered independent detections only if separated by at least 30 min; records within shorter intervals were treated as a single detection event.

We used our photographic detection dataset to characterise the temporal activity patterns of the Bornean ferret badger. We followed the statistical approach developed by Ridout and Linkie (2009) and performed all analyses in R using program Overlap v.0.3.9 (Linkie and Ridout 2011; Meredith and Ridout 2016). We defined dawn (05:00–07:00) and dusk (17:00–19:00) time periods as one‐hour pre and post sunrise/sunset, and the intervening periods as day (07:00–17:00) and night (19:00–05:00). All statistical analyses were conducted in R version 4.4.1 (R Core Team 2024).

### 2.2 Occupancy

To compare site usage among study areas, we estimated occupancy (*ψ*) of the Bornean ferret badger and the Sunda stink badger in each area where the species were detected. We constructed detection histories at the camera‐station level in R using the detectionHistory function in camtrapR v.2.3 (Niedballa et al. 2016), constructing detection histories from daily records and incorporating camera operational status so that periods during which cameras were inactive due to malfunction or downtime were treated as missing observations. To reduce data sparsity while maintaining assumptions of closure and independence, we aggregated detections into 3‐day sampling intervals. This aggregation aimed to reduce data sparsity while maintaining reasonable adherence to closure assumptions, as shorter intervals yielded sparse detection histories whereas longer intervals increased the risk of violating closure assumptions for this mobile species. We then fitted single‐season occupancy models separately for each study area using unmarked v.1.5 (Fiske and Chandler 2011), treating camera stations as replicate sampling sites. A single occupancy parameter (*ψ*) was estimated for each study area to enable robust comparisons among areas while avoiding over‐parameterisation, as detections within several areas were insufficient to reliably support occupancy models with multiple site‐level covariates. Environmental drivers of occurrence were instead evaluated using the habitat suitability modelling framework applied across all survey areas.

### 2.3 Multi‐Scale Habitat Suitability Modelling

To account for spatial clustering of detections and limitations in habitat representation associated with true absence data, we sampled pseudo‐absence points in place of observed absences when modelling habitat suitability. Although there is no evidence that the species occurs outside the Sabah highlands, we generated pseudo‐absences across the broader island to ensure systematic sampling of environmental conditions and spatial variables throughout Borneo. We generated pseudo‐absences at a 10:1 ratio relative to observed presences, following recommendations for regression‐based habitat suitability modelling using large pseudo‐absence samples to characterise available environmental space (Barbet‐Massin et al. 2012). This process was repeated 15 times, producing distinct pseudo‐absence sets, each paired with the presence data to form 15 independent presence–pseudo‐absence datasets. Potential effects of class imbalance were mitigated by averaging predictions across these independently generated datasets, and model outputs were interpreted as relative indices of habitat suitability rather than absolute occurrence probabilities.

We selected 25 spatial covariates based on their ecological relevance to southeast Asian carnivores (e.g., Hearn et al. 2018; Sartor et al. 2024), which can be categorised into six thematic groups: anthropogenic (*n* = 5), biological (*n* = 2), climatic (*n* = 5), geomorphological (*n* = 12) and land cover (*n* = 1) (Appendix S1). We implemented a three‐step, multi‐scale modelling framework following the protocol outlined by McGarigal et al. (2016) to evaluate species occurrence as a function of environmental predictors. In the initial step, each variable was assessed independently using univariate logistic regression to determine the most informative spatial scale. Predictors were examined at eight spatial extents—100 m, 250 m, 500 m, 1 km, 2 km, 4 km, 8 km and 16 km—using Gaussian kernel smoothing. The scale associated with the lowest Akaike Information Criterion (AIC; Burnham and Anderson 2003) was selected for each predictor. To maintain temporal consistency between predictor values and observation periods, environmental data were matched to the deployment year of each camera trap station. For pseudo‐absence points, predictor values were averaged across all years. All spatial datasets were harmonized to a resolution of 100 m and processed within the Google Earth Engine platform.

We evaluated multicollinearity among scale‐optimized predictors using Pearson's correlation coefficient. In cases where variables were strongly correlated (*r* ≥ 0.7), we retained the predictor with the lower associated AIC value. Each dataset was then randomly partitioned into training (80%) and testing (20%) subsets. We fitted multivariate Generalised Linear Models (GLMs) to the training sets using only the retained, uncorrelated predictors. Model selection was performed via the dredge function in the R package MuMIn v.1.48.11 (Bartoń 2025), with candidate models ranked according to ΔAIC. Model accuracy was evaluated using the Area Under the Curve (AUC) of the Receiver Operating Characteristic (ROC), calculated with the PresenceAbsence package (Freeman and Moisen 2008). All modelling steps described above (including scale selection, collinearity assessment, model fitting and evaluation) were conducted independently for each of the 15 presence–pseudo‐absence datasets. The 10 highest‐performing models (based on AUC ≥ 0.6, or ≥ 0.7 when feasible) were used to produce ensemble predictions by averaging across model outputs.

### 2.4 Extent of Occurrence and Area of Occupancy

To provide a basis for future IUCN Red List assessments of the Bornean ferret badger, we used the habitat suitability model to estimate extent of occurrence (EOO) and area of occupancy (AOO) following IUCN Red List guidelines (IUCN Standards and Petitions Committee 2024). To convert the continuous suitability surface into a binary presence–absence map, we applied a threshold based on the balance of sensitivity and specificity using ROC curves, using absences derived from our camera trap survey data. We masked clearly unsuitable areas by excluding bare granite habitat on Mount Kinabalu's summit, which is unlikely to be used by the species. The resulting binary habitat map formed the basis for AOO and EOO estimation. AOO was calculated by overlaying a 2 × 2 km grid and counting the number of cells intersecting predicted occupied habitat. EOO was calculated as the area of the minimum convex polygon (MCP) enclosing all predicted occupied cells. Spatial analyses were conducted in QGIS 3.38.1 using an equal‐area projection (EPSG:6933).

### 2.5 Population Connectivity

We applied the cumulative resistance kernel (CRK) approach (Compton et al. 2007) to delineate Bornean ferret badger core areas and factorial least cost corridors (FLCC) analysis (Cushman et al. 2009) to identify dispersal corridors. Resistance kernels model movement probability from source habitat across the landscape, producing a continuous surface that reflects movement potential throughout the area (Cushman et al. 2013). In contrast, FLCC identifies optimal pathways between pairs of source points based on minimal cumulative resistance, highlighting narrow corridors likely used for dispersal (Cushman and Landguth 2012).

We used the Connecting Landscapes (CoLa) Decision support system (Janz et al. 2025) to develop predictions of connectivity and space use. CoLa requires three core inputs, a resistance surface, population source points and a species‐specific value for maximum dispersal distance. Resistance surfaces represent the cost of moving through the landscape due to various factors including avoidance of landscape elements, physiological cost and resource availability. We generated resistance surfaces in CoLa using a negative exponential transformation of the habitat suitability layer. To account for uncertainty in the relationship between suitability and resistance, we produced four variants using shape values of 1, 4, 8 and 16. To account for increased landscape resistance to movement resulting from elevated mortality risk and other associated disturbances attributed to roads, we added a value of 30 to the centreline of pixels that lay along the main roads in our focal region. This value was selected to represent roads as partial rather than absolute barriers to movement, consistent with field observations of ferret badger detections and road mortalities recorded along these same roads, indicating reduced but not eliminated permeability. The magnitude of this resistance increment is also consistent with values applied in recent carnivore connectivity modelling studies in Sabah (e.g., Jantz et al. 2025).

We used the ‘create source points’ tool in CoLa to probabilistically distribute source points in direct proportion to values in the habitat suitability layer. We generated 1600 source points, based on a coarse population estimate derived from the area of suitable habitat within the study region (values > 0.23 in our habitat suitability layer—the same threshold used in our AOO/EOO analysis) and an estimated density of 1 individual km^−2^. While this density estimate is highly approximate, it primarily influences estimates of movement flux rather than the spatial configuration of predicted connectivity pathways; results are therefore interpreted in terms of relative connectivity patterns.

In the absence of dispersal data for the Bornean ferret badger, we developed biologically plausible estimates by applying the empirical relationship between dispersal distance and home range size, and a body mass‐based allometric model. Bowman et al. (2002) proposed that maximum dispersal distance (km) in mammals is proportional to the square root of home range area (km^2^), expressed as:

$$\text{Dispersal distance}=40\times \surd \left(\text{Home range area}\right)$$

We reviewed home range data from closely related species in the genus *Melogale*. Zhang et al. (2010) reported that Chinese ferret badgers ( *M. moschata* ) in central China had mean 100% minimum convex polygon home ranges of 128.3 ± 131.9 ha (range 51.1 to 578.2 ha). Assuming a conservative home range size of 50–100 ha for *M. everetti* , we estimate dispersal distances of 14–20 km. To cross‐validate these estimates, we applied the allometric scaling model developed by Sutherland et al. (2000), in which maximum dispersal distance (km) is estimated as:

$$D=10.9\times M^{0.44}$$

where *M* is body mass (kg). Thus, an estimated body mass range of 1–2 kg for *M. everetti* (Payne et al. 1985) yields estimated dispersal distances of 10.9–14.5 km. Given our uncertainty, we selected three different dispersal distance values to enable a sensitivity analysis, including 10, 15 and 20 km.

For the CRK we selected a linear kernel shape, ‘no’ transform and kernel volume = 1. For FLCC, we used a corridor smoothing factor of 0 and a corridor tolerance of 500 m.

## 3 Results

### 3.1 Photographic Detections and Site Occupancy and Temporal Activity

Our camera‐trap surveys yielded 407 independent photographic records of the Bornean ferret badger from 60 camera stations and 122 records of the Sunda stink badger from 22 stations across the 10 survey areas (Table 2). Ferret badgers were recorded in eight survey areas, spanning 744–2459 m elevation (mean: 1502 m). Mean detection probability per 3‐day sampling occasion was generally low across areas. Model‐based occupancy varied among survey areas and was highest in the mixed farmland area (*ψ* = 1.00 ± 0.02) and Tenompok FR (*ψ* = 0.81 ± 0.13), with similarly high values in the adjacent Kinabalu Park South (*ψ* = 0.75 ± 0.19) and moderate values in Crocker Range North (*ψ* = 0.64 ± 0.13) and Ecolinc (*ψ* = 0.49 ± 0.09). Occupancy was relatively low in Trusmadi Central (*ψ* = 0.38 ± 0.08), Kinabalu Park North (*ψ* = 0.25 ± 0.29) and Crocker Range South (*ψ* = 0.10 ± 0.05), while Bornean ferret badgers were not detected in Trusmadi (South) or Crocker Range (West). In contrast, Sunda stink badgers were recorded only in Crocker Range South (*ψ* = 0.46 ± 0.09) and Crocker Range West (*ψ* = 1.00 ± 0.01), across an elevation range of 464–1452 m. These were also the only two areas where the ferret badger showed low occupancy or was absent.

**TABLE 2:** Camera‐trap records and model‐based occupancy estimates for the Bornean ferret badger and Sunda stink badger across survey areas in Sabah's western highlands.

Region	Bornean ferret‐badger					Sunda stink badger				
	No. records	Detection probability (SD)		Occupancy (SE)		No. records	Detection probability (SD)		Occupancy (SE)	
Crocker Range (North)	51	0.07	(0.05)	0.64	(0.13)	—	—	—	—	—
Crocker Range (South)	8	0.23	(0.26)	0.10	(0.05)	119	0.12	(0.05)	0.46	(0.09)
Crocker Range (West)	—	—	—	—	—	3	0.01	(0.02)	1.00	(0.01)
Ecolinc (Mosaic)	149	0.16	(0.05)	0.49	(0.09)	—	—	—	—	—
Kinabalu Park (North)	1	0.05	(0.10)	0.25	(0.29)	—	—	—	—	—
Kinabalu Park (South)	45	0.14	(0.20)	0.75	(0.19)	—	—	—	—	—
Mixed farmland	5	0.02	(0.04)	1.00	(0.02)	—	—	—	—	—
Tenompok FR	69	0.22	(0.10)	0.81	(0.13)	—	—	—	—	—
Trusmadi FR (Central)	79	0.08	(0.02)	0.38	(0.08)	—	—	—	—	—
Trusmadi FR (South)	—	—	—	—	—	—	—	—	—	—

*Note:* ‘No. records’ indicates the number of independent photographic detections within each study area. Detection probability (*p*) represents the mean predicted probability of detection per 3‐day sampling occasion, conditional on occupancy (values in parentheses show standard deviation among camera stations). Occupancy (*ψ*) represents the estimated proportion of camera stations occupied within each area (standard errors in parentheses). Dashes indicate areas where the species was not detected.

Camera‐trap data indicated that the Bornean ferret badger was strongly nocturnal, with only 3 of 407 detections occurring during daylight hours (Appendix S2). Activity was distributed throughout the nocturnal period, peaking during the 3 h preceding dawn before declining sharply at sunrise.

### 3.2 Multi‐Scale Habitat Modelling

To facilitate interpretation, here we refer to selected spatial scales as fine (100–250 m), moderate (500 m–2 km) and broad (4–16 km). The GLM analysis identified habitat associations primarily with broad‐scale predictors, with moderate‐scale effects less frequent and fine‐scale variables rarely retained. Model coefficients and scale selection for all retained predictors are provided in Appendix S3 and summarised in Appendix S4. Across models, the most consistent effects were observed for topographic position index, water availability and tree cover. Topographic position index showed positive associations at moderate scales, indicating higher suitability on gently convex landforms such as upper slopes and ridgelines. Water availability exhibited consistent negative associations at broad scales, suggesting reduced suitability in wetter valley environments. Tree cover showed weak but consistent negative associations at broad scales, indicating slightly higher suitability in more heterogeneous or partially open forest landscapes.

Additional predictors showed weaker or less consistent effects. Accessibility appeared in seven models and was positively associated at broad scales. Precipitation, also retained in seven models, showed weak negative associations at broad scales, while precipitation seasonality was included in five models with weak positive associations at moderate scales. Soil organic carbon was retained in seven models and showed weak positive associations at fine and moderate scales, while soil nitrogen appeared in four models with weak positive associations at broad scales. The global human modification index appeared in three models with positive associations at broad scales, and soil sand showed weak negative associations at moderate scales. Land‐cover predictors were less frequently retained: deforested tropical moist forest appeared in two models with mixed effects, while non‐forest land‐cover types were negatively associated at fine scales. Soil cation exchange capacity and soil clay were each retained in two models with weak and inconsistent effects at broad scales.

**FIGURE 2:**
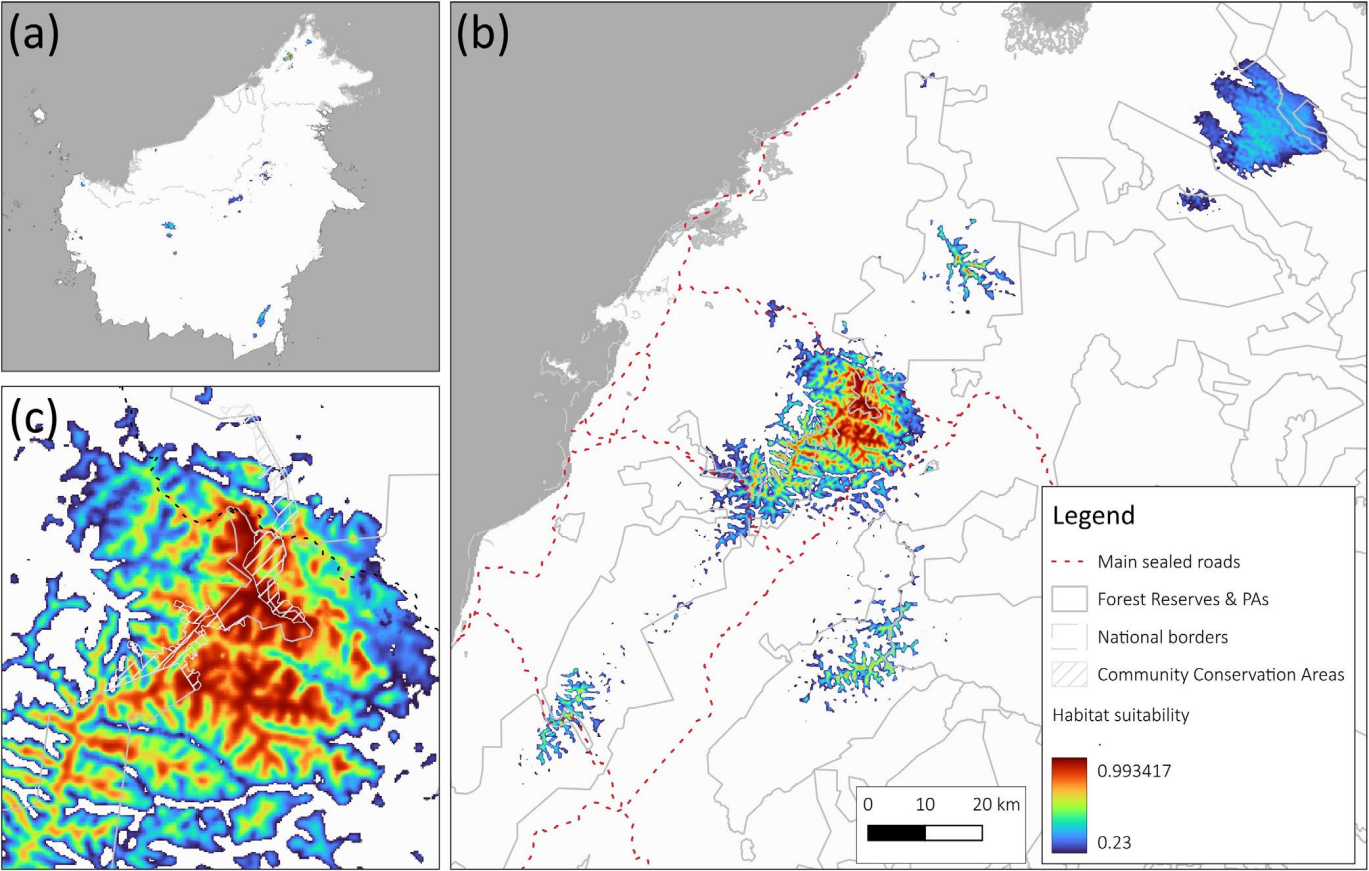
Ensemble habitat suitability model for the Bornean ferret badger, showing three different focal areas: (a) Borneo, (b) western Sabah, (c) Kinabalu Ecolinc.

The predicted habitat suitability surface revealed three clusters of moderate to high suitability on the island of Borneo (Figure 2); however, predicted suitability outside Sabah represents model‐based projections and remains hypothetical in the absence of confirmed records. The highest region of suitability was located in parts of north and northwestern Sabah; the second included patches of moderate suitability across Borneo's mountainous interior, encompassing the Kelabit Uplands and Kayan Mentarang region, as well as part of interior West Kalimantan; and the third was situated in the Meratus Mountains of South Kalimantan. Outside of these areas, predicted suitability was negligible, with the vast majority of the landscape showing very low or no predicted suitability. As the species has only ever been recorded in the northwestern highlands of Sabah, we focus our discussion of results exclusively on this region.

In Sabah, the largest and highest predicted area of suitability was centred on Tenompok FR and the Kinabalu Ecolinc, extending into the southernmost portion of Kinabalu Park and the northern extremity of Crocker Range Park. This core area is separated from three smaller patches of moderate suitability within the broader Crocker Mountain Range: one located in the high‐elevation zone of central Crocker Range Park, straddling the Keningau–Kota Kinabalu road, another in central Kinabalu Park, encompassing the peaks in and around Gunung Nambuyukong. A third patch was identified within central Nuluhon‐Trusmadi Forest Reserve, encompassing the reserve's highest peaks and associated high‐elevation ridgelines, including Gunung Trusmadi, Sinsing and Kaingaran. Lastly, a region of low suitability was identified in the Beluran District, centred on moderately elevated terrain including parts of the Paitan Forest Reserve.

### 3.3 Extent of Occurrence and Area of Occupancy

We applied a threshold of 0.23 to the habitat suitability model, selecting this value based on ROC curve analysis to optimise the balance between sensitivity and specificity. At this threshold, the model performed well, with a mean AUC of 0.79 ± 0.03, sensitivity of 0.91 ± 0.04, specificity of 0.58 ± 0.04 and an overall classification accuracy (PCC) of 0.68 ± 0.03. Based on the number of 2 × 2 km grid cells intersecting predicted occupied habitat, we calculated an Area of Occupancy (AOO) of 2424 km^2^. We calculated the Extent of Occurrence (EOO) as 4795.55 km^2^, using the area of the minimum convex polygon enclosing all predicted occupied habitat patches. Estimates of occupied area depend on threshold selection and should therefore be interpreted as approximate; however, small threshold changes do not alter the overall spatial pattern of predicted occupied habitat or the study's conservation conclusions.

### 3.4 Population Connectivity

**FIGURE 3:**
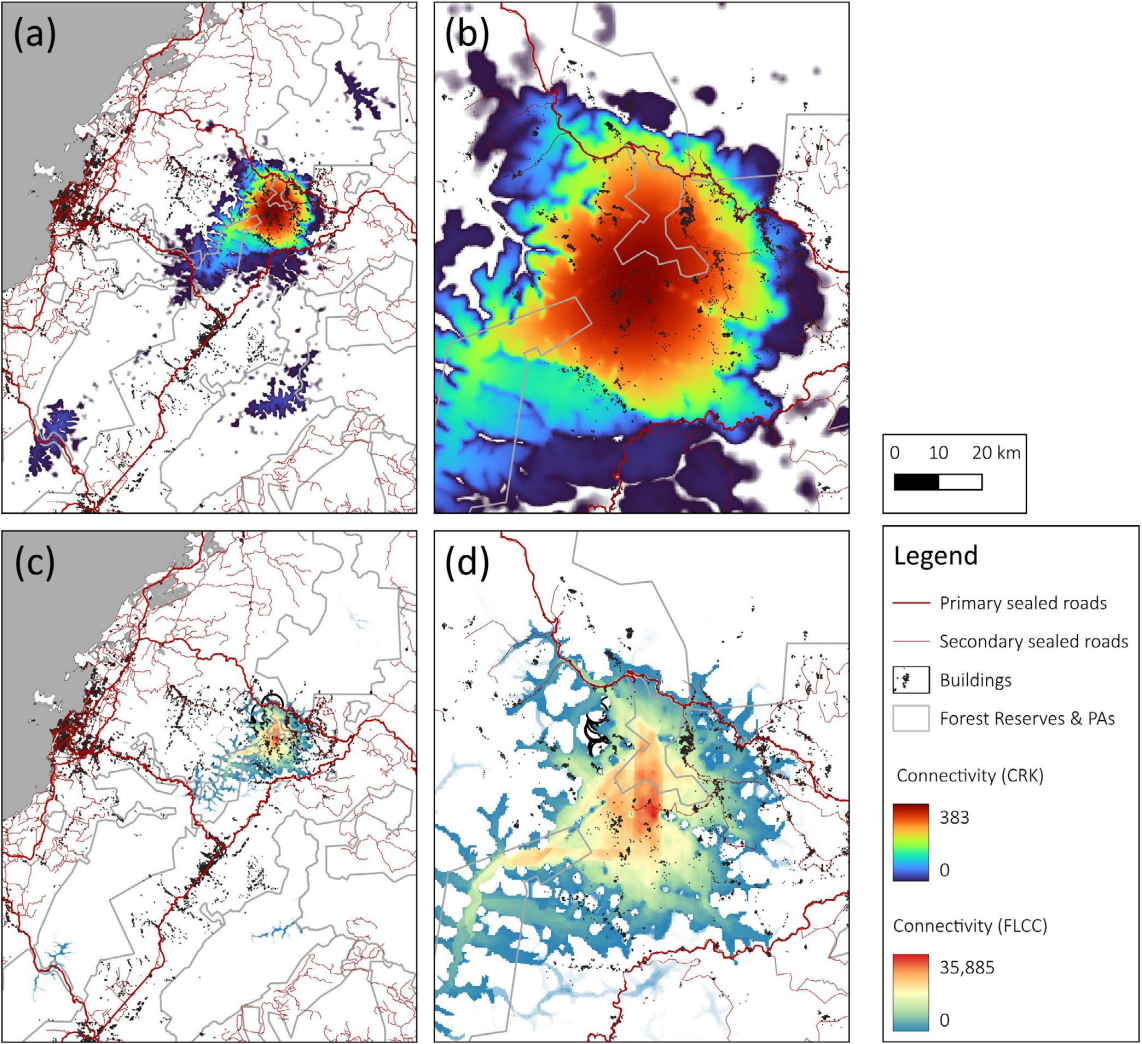
Predicted population connectivity for the Bornean ferret badger in Sabah's western highlands based on cumulative resistance kernel (CRK) and factorial least‐cost corridor (FLCC) analyses under an intermediate dispersal and resistance parameterisation (15 km dispersal distance; conversion shape parameter = 8). Panels (a) and (c) show regional patterns of relative movement potential across the western highlands, while panels (b) and (d) provide zoomed views centred on the Kinabalu Ecolinc landscape. Panels (a, b) depict CRK outputs and panels (c, d) depict FLCC outputs.

Both the CRK and FLCC analyses identified four spatially discrete zones of high movement potential within highly suitable habitat patches in Sabah's western highlands. Under an intermediate dispersal and resistance parameterisation (15 km dispersal distance; conversion shape parameter = 8), the largest core encompassed the Tenompok FR–Kinabalu Ecolinc landscape, extending into northern Crocker Range Park and the southern extremity of Kinabalu Park, and covered approximately 500 km^2^ (Figure 3). Three smaller cores were delineated in southern Crocker Range Park, central Kinabalu Park and the central Nuluhon–Trusmadi Forest Reserve, each remaining < 100 km^2^. Both connectivity metrics indicated largely continuous movement potential within habitat patches, but no inter‐patch connectivity was predicted. Neighbouring habitat patches were separated by approximately 21–35 km of lower‐suitability landscape, with edge‐to‐edge distances of ~21 km between Tenompok/Kinabalu–Ecolinc and central Kinabalu Park, ~25 km to Nuluhon–Trusmadi, and ~35 km to southern Crocker Range. Sensitivity analyses across alternative dispersal distances and resistance‐surface parameterisations produced comparable spatial configurations, with the same four habitat cores consistently identified (Appendixes S5 and S6). However, estimated core area increased substantially from the most conservative to the most permissive scenarios, indicating that while overall patch structure was robust, estimates of core extent were sensitive to assumptions regarding dispersal ability and landscape resistance. No scenario predicted functional corridors between habitat cores.

## 4 Discussion

This study presents the most comprehensive assessment of the Bornean ferret badger's distribution and habitat associations to date, integrating targeted camera‐trap surveys with spatial modelling. Among the most significant outcomes was the first confirmed record of the species in the Nuluhon–Trusmadi Forest Reserve, extending its known range eastward into the Trusmadi massif and providing the first verified records beyond the Crocker–Kinabalu area.

Model‐based occupancy estimates were relatively high in Tenompok Forest Reserve and adjacent areas of the Kinabalu Ecolinc and southern Kinabalu Park, spanning a mosaic of habitats with varied disturbance histories—including selectively logged forest, regenerating secondary forest and undisturbed upland forest. Our data suggest that the Bornean ferret badger not only tolerates but may benefit from certain forms of disturbance. Occupancy was comparatively high in selectively logged Tenompok and regenerating Ecolinc forest relative to several adjacent primary‐forest sites. This pattern is broadly consistent with the weak but consistent negative association between occurrence and forest cover identified in the habitat suitability models. Although the mechanisms may differ—e.g., enhanced understorey complexity in logged forest vs. elevated soil fertility and prey biomass in regenerating fields—both may create structurally heterogeneous conditions that improve foraging opportunities. These findings echo Nagano et al. (2019), who recorded the species in open slash‐and‐burn fields, including signs of burrow use, but not in secondary forest nearby. This broad local habitat use contrasts sharply with the species' narrow regional range, raising an apparent ecological paradox: how can a species occupy diverse habitats locally yet remain geographically restricted?

Multi‐scale habitat suitability modelling identified several predictors of occurrence operating primarily at moderate to broad spatial scales. Topographic position index was among the strongest, suggesting higher suitability on gently convex landforms—such as upper slopes and ridgelines—over valley bottoms. While valleys may offer greater organic content, upper slopes may offer more stable and well‐drained substrates for fossorial activity. Soil organic carbon and nitrogen were also positively associated with occurrence, potentially reflecting increased invertebrate prey availability (e.g., earthworms; Payne et al. 1985). This combination of landform and edaphic preferences may be unevenly distributed across Borneo, partially explaining why *M. everetti* occupies diverse habitat types locally, yet remains regionally restricted.

Intraguild competition may further contribute to this pattern. The Sunda stink badger is a widespread, disturbance‐tolerant musteloid, recorded across a wide range of habitats in Borneo—including lowland forest, logged forest and oil palm (Samejima et al. 2016; Ross et al. 2017). In contrast, we detected Mydaus in only two of ten areas across the western highlands—Crocker Range (South) and Crocker Range (West)—which were also the only areas where *M. everetti* was absent or occurred at notably low occupancy. This consistent spatial segregation is consistent with the hypothesis that competitive interactions may contribute to the realised distribution of the species. The two species are similar in size, diet and fossorial behaviour, and may compete for shared trophic resources in areas of overlap. *M. everetti* may thus be restricted to elevations or soil types where niche overlap is reduced and competition is less intense. Although this pattern is consistent with the hypothesis that interspecific competition may contribute to the Bornean ferret badger's narrow realised range, historical biogeographic processes and unmeasured environmental gradients may also play a role. Further work—including dietary analysis, behavioural observations and spatial co‐occurrence modelling—could help test this hypothesis.

Our results reinforce the conclusion that the Bornean ferret badger is confined to the north‐western montane region of Borneo, with all confirmed detections from the Kinabalu–Crocker–Trusmadi complex. While our models predicted additional areas of moderate suitability—in the Kelabit Highlands, Kayan Mentarang, interior West Kalimantan and the Meratus Mountains—these areas remain unsurveyed or have yielded no records despite targeted carnivore surveys (Wilting et al. 2016; Brodie et al. 2015). Predicted suitability outside surveyed regions should therefore be interpreted cautiously, as models are based on environmental similarity rather than confirmed species' absence. Although estimated detection probability was generally low, repeated camera‐trap deployment resulted in consistent detection where the species was present, and so this absence is unlikely to reflect poor detectability alone. The species is probably either absent or occurs at very low density in these regions. Nonetheless, targeted surveys in the interior uplands would be valuable for evaluating model predictions and refining range boundaries.

Our camera‐trap data show that Bornean ferret badgers can persist—and potentially thrive—in regenerating forest shaped by traditional swidden agriculture, suggesting that historical land‐use patterns in Ecolinc do not currently impede movement. However, this landscape is under rapid transition. Expansion of towns along the Pan Borneo Highway, the construction of the highway itself, and the upgraded Keningau–Ranau road—which now separates Kinabalu–Crocker populations from those in Nuluhon–Trusmadi—may further fragment habitat and isolate subpopulations. Given the species' likely limited dispersal ability, this raises concerns over genetic isolation. Future research should assess population connectivity using genetic data and explore the permeability of these modified landscapes to movement.

We estimated an AOO of 2424 km^2^ and an EOO of 4795.6 km^2^. These figures exceed those in the current IUCN Red List (Wilting et al. 2015), largely due to new records and improved spatial resolution, including the addition of Nuluhon–Trusmadi. Although Bornean ferret badgers appear to be confined to a narrow region, our results suggest that suitable habitat within this range appears to be fragmented into discrete patches separated by areas of lower suitability. Connectivity modelling identified four core areas with internally contiguous movement zones but no functional corridors between them.

Our results have direct implications for biodiversity conservation in Sabah's montane landscapes. Tenompok Forest Reserve, the Kinabalu Ecolinc and adjacent portions of Kinabalu Park emerge as key core areas for the Bornean ferret badger and should be prioritised for long‐term protection and management. The absence of functional corridors among core habitat patches highlights the potential isolating effects of recent and planned road infrastructure, particularly along the Keningau–Ranau axis, and underscores the need for targeted mitigation measures. Maintaining and expanding community‐managed forest mosaics within the Kinabalu Ecolinc is likely to be critical for sustaining connectivity. More broadly, our findings illustrate how fine‐scale environmental filters and landscape structure can constrain the distributions of endemic carnivores, even where local habitat tolerance appears high.

### 4.1 Study Limitations and Survey Priorities

While this study provides the most extensive dataset on *M. everetti* to date, survey coverage was limited by logistical and financial constraints. Camera‐trap effort was concentrated in a subset of sites, leaving parts of both high‐ and low‐suitability habitat unsampled. Kinabalu Park remains under‐surveyed despite predicted heterogeneity in habitat suitability. Human‐dominated landscapes separating the Ecolinc from Nuluhon–Trusmadi also require targeted effort to determine whether they function as barriers or corridors. Expanding coverage across these and other areas will be essential to refine range limits, identify potential corridors and confirm additional populations.

### 4.2 Conservation Outlook

This highly restricted status, paired with its ecological distinctiveness and tolerance of traditional upland land‐use mosaics, gives the Bornean ferret badger strong potential as a flagship for the conservation of montane biodiversity in northern Borneo. Promotion of a regionally resonant common name, such as ‘Kinabalu ferret badger’, could raise public awareness. Its presence in the Kinabalu Ecolinc corridor also opens the door to sustainable ecotourism, potentially benefiting local communities through mammal‐watching initiatives. However, our fieldwork also revealed direct anthropogenic pressures. *M. everetti* is sometimes trapped and consumed opportunistically by villagers, and we documented instances of domestic dogs killing individuals. These threats, although likely incidental, could amplify the impacts of habitat fragmentation. The species also remains formally listed under the outdated name *Melogale personata* in the Sabah Wildlife Conservation Enactment (1997), creating ambiguity in legal protection. Updating its legal status and improving community engagement will be critical steps in safeguarding this evolutionarily distinct carnivore—one that, as far as current evidence suggests, may be found nowhere else but Sabah.

## Author Contributions

**Andrew J. Hearn:** conceptualization (lead), data curation (equal), formal analysis (equal), funding acquisition (equal), investigation (equal), methodology (equal), project administration (equal), visualization (lead), writing – original draft (lead), writing – review and editing (lead). **Mohammad Aliyuddin bin Jaini:** data curation (equal), investigation (equal), project administration (equal), writing – review and editing (supporting). **Caroline Charão Sartor:** formal analysis (equal), methodology (equal), writing – review and editing (supporting). **Pg Mohd Sahlan bin Salam:** writing – review and editing (supporting). **Andy Martin:** writing – review and editing (supporting). **Chrishen Gomez:** formal analysis (equal), methodology (equal), writing – review and editing (supporting). **David W. Macdonald:** funding acquisition (equal), writing – review and editing (supporting).

## Funding

This work was supported by the Robertson Foundation, Point Defiance Zoo & Aquarium's Dr. Holly Reed Conservation Fund and Panthera's Small Cat Action Fund.

## Conflicts of Interest

The authors declare no conflicts of interest.

## Supporting information


**Appendix S1:** Table showing covariates used in the multi‐scale habitat suitability modelling.
**Appendix S2:** Bornean ferret badger temporal activity.
**Appendix S3:** Table showing predictor variables, spatial scales, coefficients and associated *p*‐values for the 10 highest‐performing Generalised Linear Models (GLMs) used to develop the ensemble habitat suitability model for the Bornean ferret badger ( *Melogale everetti* ). Models were fitted to presence–pseudo‐absence datasets following multi‐scale optimisation.
**Appendix S4:** Table showing summary of predictor variables retained in the 10 highest‐performing presence–pseudo‐absence Generalised Linear Models (GLMs) used to construct the final ensemble habitat suitability model for the Bornean ferret badger ( *Melogale everetti* ).
**Appendix S5:** Maps showing Bornean ferret badger Cumulative resistant kernels developed using 3 different maximum dispersal distances and 4 different resistant shapes.
**Appendix S6:** Maps showing Bornean ferret badger factorial least cost corridors developed using 3 different maximum dispersal distances and 4 different resistant shapes.

## Data Availability

Due to the conservation sensitivity of this threatened species and in accordance with local regulatory restrictions, precise spatial location data cannot be made publicly available. Non‐spatial datasets used in the occupancy analysis, including detection histories and survey effort information, are provided as Supporting Information. Additional derived data supporting the findings of this study are available for scholarly use upon reasonable request from the corresponding author.
