# Supplementary material for: Distribution, Habitat Use and Conservation of the Bornean Ferret Badger
Source: Ecol Evol. 2026 Jun 3;16(6):e73756. doi: 10.1002/ece3.73756 (PMC13239073; doi:10.1002/ece3.73756)

# Appendix

Appendix S1. Table showing covariates used in the multi-scale habitat suitability modelling

Appendix S2. Bornean ferret badger temporal activity.


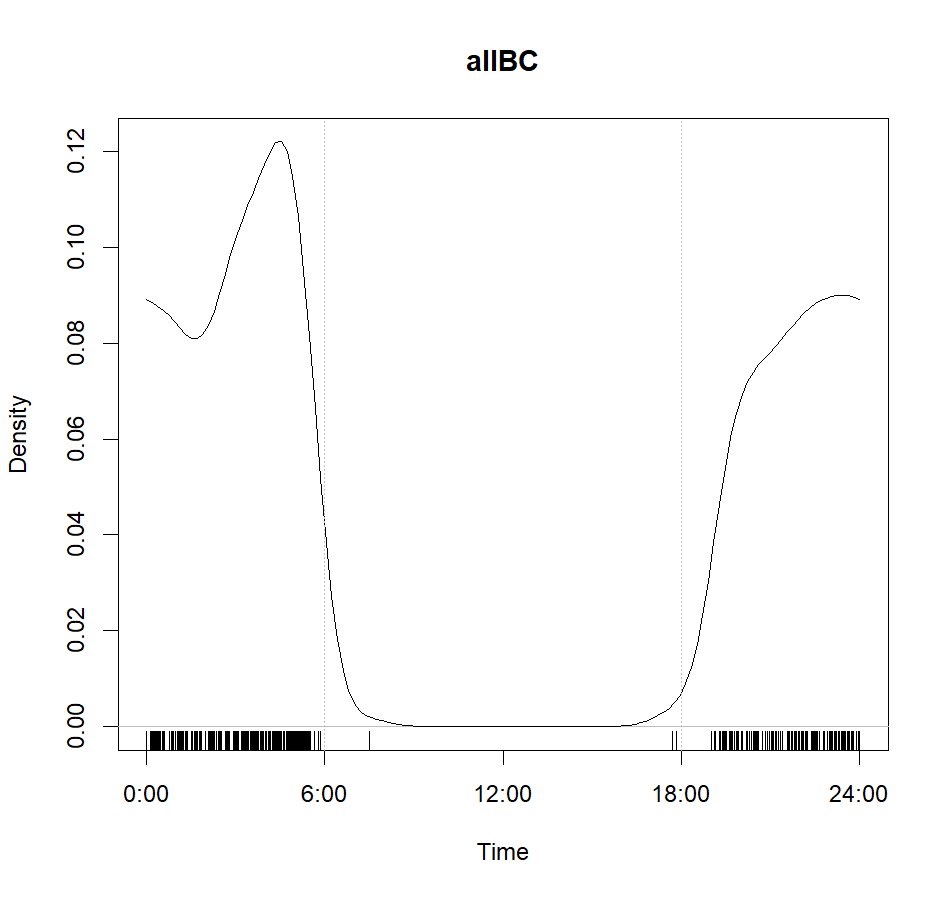


Appendix S3. Table showing predictor variables, spatial scales, coefficients, and associated p-values for the ten highest-performing Generalised Linear Models (GLMs) used to develop the ensemble habitat suitability model for the Bornean ferret badger (Melogale everetti). Models were fitted to presence–pseudo-absence datasets following multi-scale optimisation.

| Model | Variable | Spatial scale (m) | Coefficient | p |
| --- | --- | --- | --- | --- |
| FB_pseudo_1_10 | (Intercept) | NA | 233.79 | 0.003 |
|  | Accessibility | 16,000 | 217.89 | 0.000 |
|  | Precipitation | 16,000 | -0.04 | 0.004 |
|  | Soil_BDOD | 16,000 | -0.84 | 0.001 |
|  | Soil_Sand | 2,000 | -0.07 | 0.165 |
|  | TPI | 500 | 1.01 | 0.001 |
|  | Tree_Cover | 16,000 | -0.58 | 0.002 |
|  | Water | 16,000 | -186.11 | 0.001 |
|  |  |  |  |  |
| FB_pseudo_1_10_model2 | (Intercept) | NA | -1.67 | 0.913 |
|  | Accessibility | 8,000 | 282.15 | 0.000 |
|  | Land cover: deforested tropical moist forest | 16,000 | 35.23 | 0.014 |
|  | Land cover: other | 250 | -18.38 | 0.018 |
|  | Precipitation | 16,000 | -0.02 | 0.006 |
|  | Soil_Nitrogen | 16,000 | 0.01 | 0.003 |
|  | Soil_Sand | 1,000 | -0.08 | 0.027 |
|  | Soil_SOC | 500 | 0.01 | 0.006 |
|  | TPI | 500 | 0.86 | 0.001 |
|  | Water | 8,000 | -269.26 | 0.001 |
|  |  |  |  |  |
| FB_pseudo_1_10_model3 | (Intercept) | NA | 63.25 | 0.010 |
|  | Accessibility | 16,000 | 214.42 | 0.000 |
|  | Land cover: deforested tropical moist forest | 16,000 | -33.51 | 0.024 |
|  | Land cover: degraded tropical moist forest | 4,000 | 17.24 | 0.008 |
|  | Land cover: other | 250 | -7.25 | 0.083 |
|  | Precipitation | 16,000 | -0.02 | 0.016 |
|  | Precipitation_seasonality | 2,000 | 1.53 | 0.002 |
|  | Soil_SOC | 500 | 0.01 | 0.005 |
|  | TPI | 500 | 0.44 | 0.008 |
|  | Trap_night | NA | 0.03 | 0.094 |
|  | Tree_Cover | 16,000 | -0.85 | 0.000 |
|  | Water | 16,000 | -178.23 | 0.000 |
|  |  |  |  |  |
| FB_pseudo_1_10_model4 | (Intercept) | NA | -7.94 | 0.058 |
|  | Accessibility | 16,000 | 123.19 | 0.000 |
|  | Precipitation | 16,000 | -0.01 | 0.000 |
|  | Soil_SOC | 500 | 0.01 | 0.000 |
|  | TPI | 500 | 0.25 | 0.005 |
|  | Tree_Cover | 16,000 | 0.13 | 0.000 |
|  |  |  |  |  |
| FB_pseudo_1_10_model5 | (Intercept) | NA | 32.40 | 0.059 |
|  | Accessibility | 16,000 | 108.85 | 0.008 |
|  | Precipitation | 16,000 | -0.03 | 0.001 |
|  | Precipitation_seasonality | 2,000 | 1.37 | 0.001 |
|  | Soil_CEC | 16,000 | 0.14 | 0.000 |
|  | TPI | 500 | 0.48 | 0.001 |
|  | Tree_Cover | 16,000 | -0.50 | 0.001 |
|  | Water | 16,000 | -130.08 | 0.000 |
|  |  |  |  |  |
| FB_pseudo_1_10_model6 | (Intercept) | NA | -7.66 | 0.221 |
|  | GModificationIndex | 16,000 | 104.24 | 0.000 |
|  | Precipitation_seasonality | 2,000 | 0.45 | 0.011 |
|  | Soil_SOC | 500 | 0.01 | 0.002 |
|  | TPI | 500 | 0.43 | 0.004 |
|  | Tree_Cover | 16,000 | -0.39 | 0.000 |
|  | Water | 16,000 | -138.67 | 0.000 |
|  |  |  |  |  |
| FB_pseudo_1_10_model7 | (Intercept) | NA | -69.76 | 0.000 |
|  | GModificationIndex | 16,000 | 36.24 | 0.013 |
|  | Precipitation_seasonality | 2,000 | 0.36 | 0.011 |
|  | Soil_Clay | 16,000 | 0.12 | 0.000 |
|  | Soil_Nitrogen | 16,000 | 0.00 | 0.001 |
|  | Soil_SOC | 100 | 0.01 | 0.002 |
|  | TPI | 500 | 0.31 | 0.012 |
|  | Tree_Cover | 16,000 | -0.34 | 0.001 |
|  | Water | 8,000 | -112.36 | 0.002 |
|  |  |  |  |  |
| FB_pseudo_1_10_model11 | (Intercept) | NA | -51.90 | 0.000 |
|  | GModificationIndex | 16,000 | 68.78 | 0.000 |
|  | Land cover: forest regrowth | 4,000 | 28.01 | 0.011 |
|  | Soil_CEC | 16,000 | 0.11 | 0.003 |
|  | Soil_SOC | 500 | 0.01 | 0.000 |
|  | Water | 8,000 | -136.01 | 0.000 |
|  |  |  |  |  |
| FB_pseudo_1_10_model12 | (Intercept) | NA | 25.27 | 0.189 |
|  | Accessibility | 16,000 | 323.94 | 0.000 |
|  | Precipitation | 16,000 | -0.03 | 0.020 |
|  | Soil_Nitrogen | 16,000 | 0.01 | 0.004 |
|  | Soil_Sand | 1,000 | -0.05 | 0.099 |
|  | Soil_SOC | 100 | 0.01 | 0.017 |
|  | Temperature_seasonality | 16,000 | 10.72 | 0.010 |
|  | TPI | 500 | 0.45 | 0.007 |
|  | Tree_Cover | 16,000 | -0.25 | 0.039 |
|  | Water | 16,000 | -189.94 | 0.000 |
|  |  |  |  |  |
| FB_pseudo_1_10_model15 | (Intercept) | NA | 47.19 | 0.040 |
|  | Accessibility | 16,000 | 158.26 | 0.002 |
|  | Precipitation | 16,000 | -0.02 | 0.001 |
|  | Precipitation_seasonality | 2,000 | 0.96 | 0.002 |
|  | Soil_Clay | 16,000 | -0.08 | 0.128 |
|  | Soil_Nitrogen | 16,000 | 0.01 | 0.000 |
|  | TPI | 500 | 0.27 | 0.039 |
|  | Tree_Cover | 16,000 | -0.24 | 0.033 |
|  | Water | 16,000 | -92.64 | 0.000 |

Appendix S4. Table showing summary of predictor variables retained in the ten highest-performing presence–pseudo-absence Generalised Linear Models (GLMs) used to construct the final ensemble habitat suitability model for the Bornean ferret badger (*Melogale everetti*).

Appendix S5. Maps showing Bornean ferret badger Cumulative resistant kernels developed using 3 different maximum dispersal distances and 4 different resistant shapes.


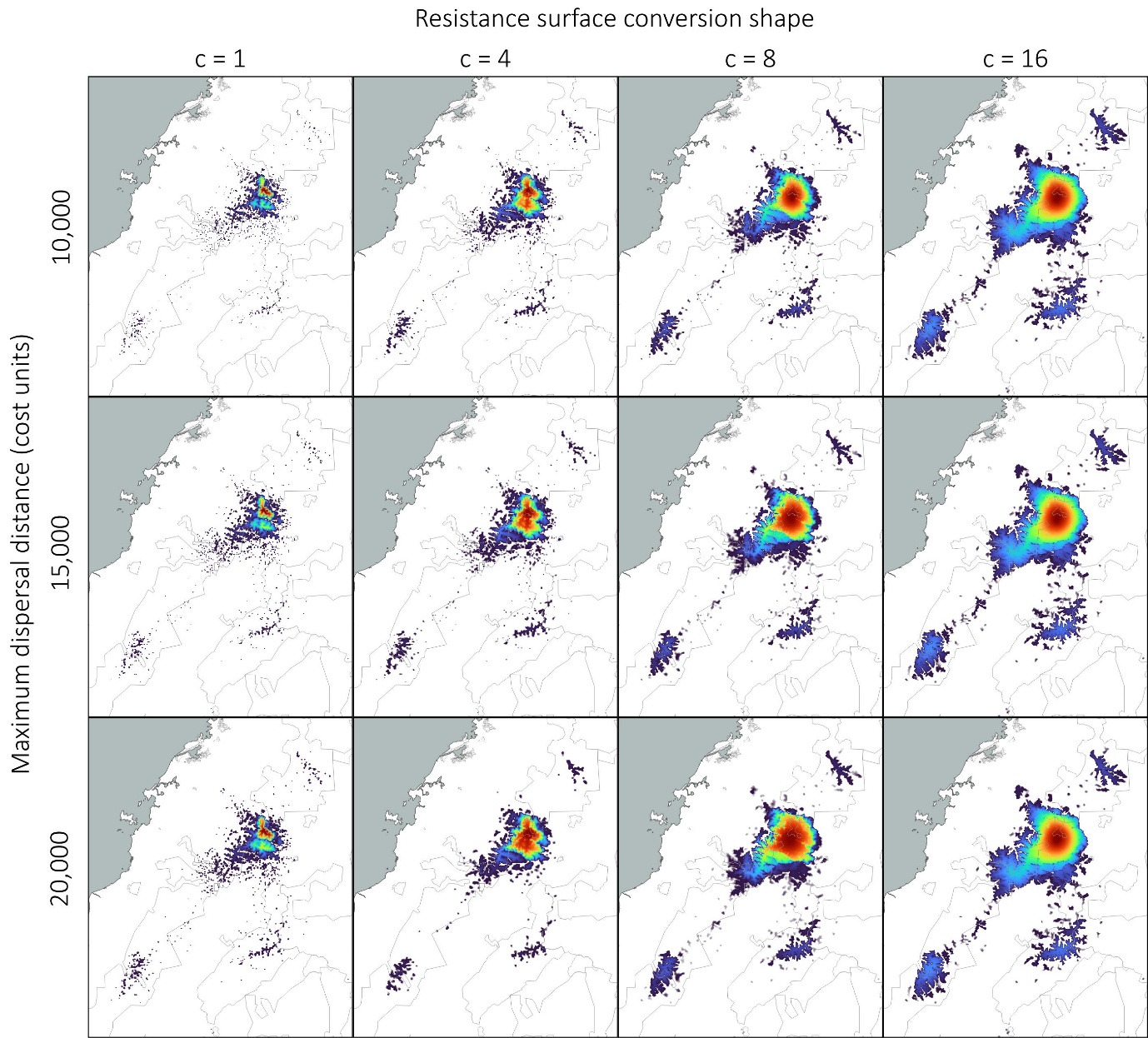


Appendix S6. Maps showing Bornean ferret badger factorial least cost corridors developed using 3 different maximum dispersal distances and 4 different resistant shapes.


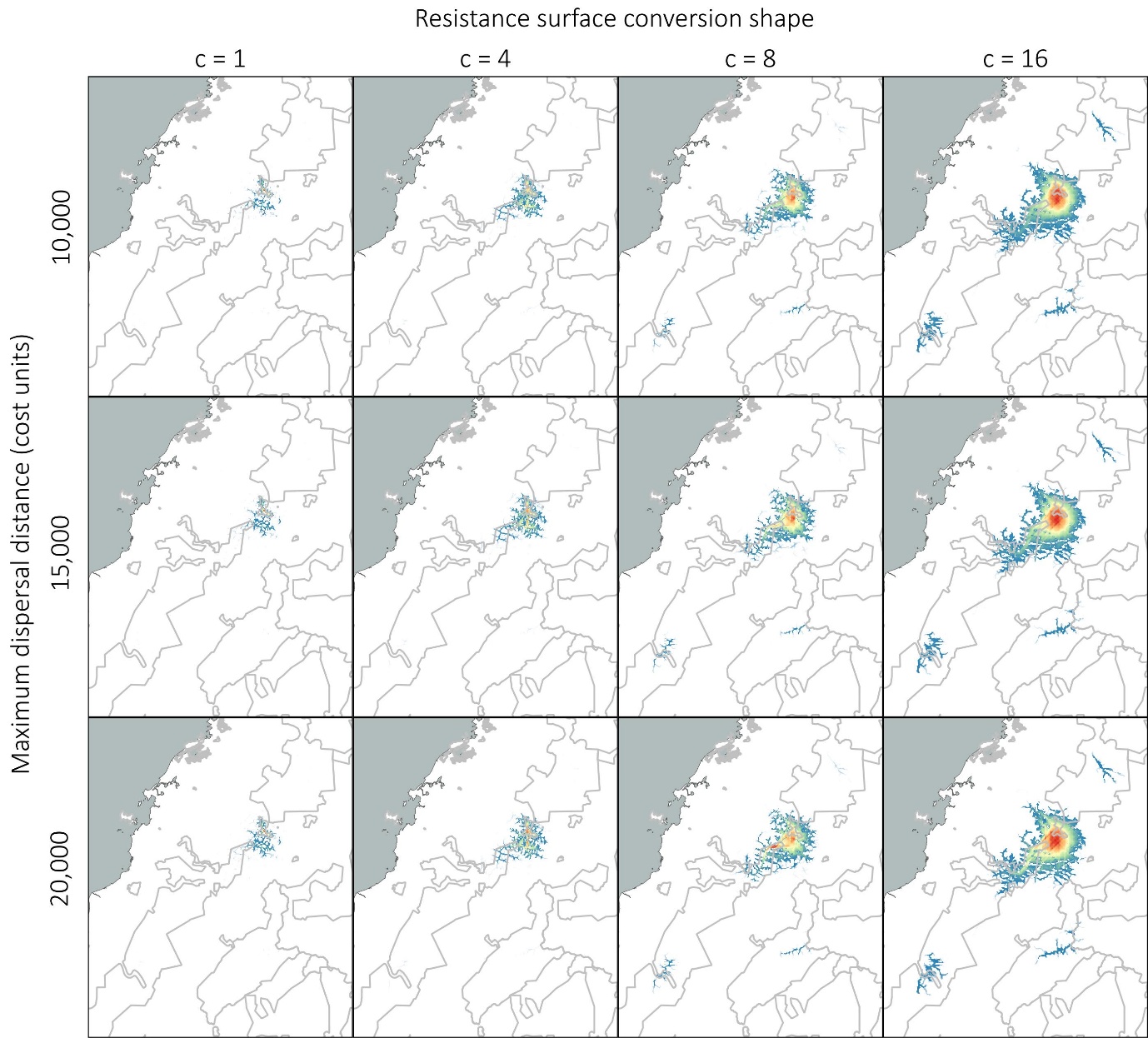

Supplement: Supplementary file 1 — Appendix S1: Table showing covariates used in the multi‐scale habitat suitability modelling. Appendix S2: Bornean ferret badger temporal activity. Appendix S3: Table showing predictor variables, spatial scales, coefficients and associated p‐values for the 10 highest‐performing Generalised Linear Models (GLMs) used to develop the ensemble habitat suitability model for the Bornean ferret badger ( Melogale everetti ). Models were fitted to presence–pseudo‐absence datasets following multi‐scale optimisation. Appendix S4: Table showing summary of predictor variables retained in the 10 highest‐performing presence–pseudo‐absence Generalised Linear Models (GLMs) used to construct the final ensemble habitat suitability model for the Bornean ferret badger ( Melogale everetti ). Appendix S5: Maps showing Bornean ferret badger Cumulative resistant kernels developed using 3 different maximum dispersal distances and 4 different resistant shapes. Appendix S6: Maps showing Bornean ferret badger factorial least cost corridors developed using 3 different maximum dispersal distances and 4 different resistant shapes. [file ECE3-16-e73756-s001.docx]
